# Supplementary material for: Deep-learning-based image super-resolution of an end-expandable optical fiber probe for application in esophageal cancer diagnostics
Source: J Biomed Opt. 2024 Apr 4;29(4):046001. doi: 10.1117/1.JBO.29.4.046001 (PMC10993061; doi:10.1117/1.JBO.29.4.046001)
Supplement: Supplementary file 1 [file JBO_029_046001_SD001.pdf]

Deep learning-based image super-resolution of a novel end-expandable optical fiber probe for application in esophageal cancer diagnostics

Xiaohui Zhang,[a] Mimi Tan,[b] Mansour Nabil,[b] Richa Shukla,[b] Shaleen Vasavada,[b] Sharmila Anandasabapathy,[b,c] Mark A. Anastasio,[a*] Elena Petrova[b,c*]

[a]Department of Bioengineering, University of Illinois Urbana-Champaign, Urbana, IL, USA 61801
[b]Section of Gastroenterology and Hepatology, Department of Medicine, Baylor College of Medicine, TX, USA 77030
[c]Baylor Global Health, Baylor College of Medicine, TX, USA

SUPPLEMENTAL MATERIAL

Prototyping the optical fiber brush

To prove the feasibility of the optical fiber brush prototyping we tested our hypothesis using commercially available unfused optical fibers (see Figure S1.)

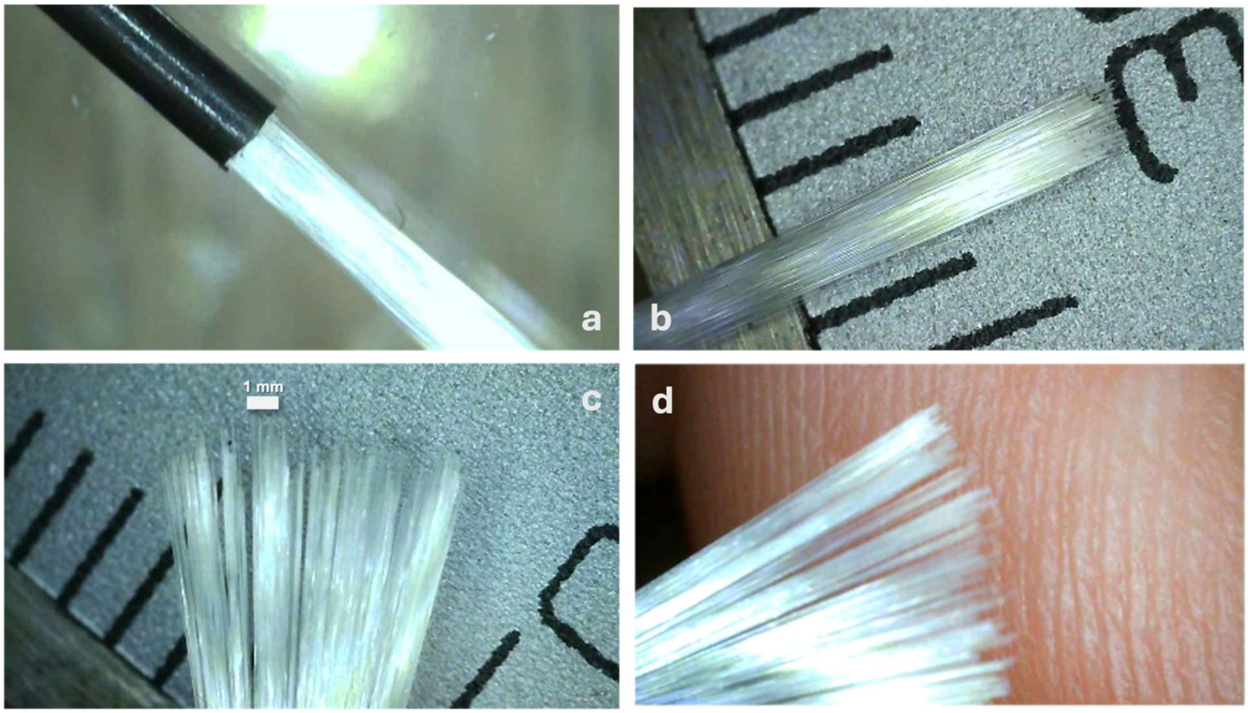

Figure S1. (a) A view of optical fiber bundle (Schott Inc.) with partially removed black cladding. The outer diameter of the glass fiber bundle is approximately 600 μm, and the diameter of a single fiber is 29 μm. (b, c) The unfused fiber bundle is shown with a scale bar, and the same fiber bundle is fanned. (d) Contact of the optical fiber brush with finger skin is a safe manner (Video 1, MP4, 6.2 MB).

We used optical fiber bundles provided by Schott Inc. as they are produced without fusion along the length of the fiber bundle. These fiber bundles were only fused at the ends. Note that individual microfibers can be easily separated along the fiber bundle when cut off at about an inch from the ends. In the experiment, we used two types of optical fiber bundles with outer diameters of 500 μm and 600 μm and with the diameters of single microfibers of 6 μm and 29 μm, respectively.

To demonstrate the flexibility of single fibers, we fanned the free end of the fiber bundle and recorded a video showing a fanned fiber bundle that gently touched finger skin (Figure S1). It is important that breakage of the flexible individual fiber was not observed for the tested fibers. In fact, the examined tissue surface is never flat and may even benefit from the shape of the probe. The radius of curvation of glass fiber bundles with the same material properties depends on their cross-sections. In general, the smaller cross-section of a fiber the more it can be bent. While an ordinary fiber bundle has a very high radius of curvation, a microfiber exhibits features of unexpected flexibility.

We did not observe spontaneously broken microfibers in our experiments. In the future, the safety of the optical fiber brush in a simulated endoscopic environment must be tested with more accuracy, and potential collaboration with optical fiber manufacturers to improve and ensure the safety of the optical fiber brush will be considered. In summary, our current observations demonstrated that the optical fiber brush prototype with an expanded end can be used safely.

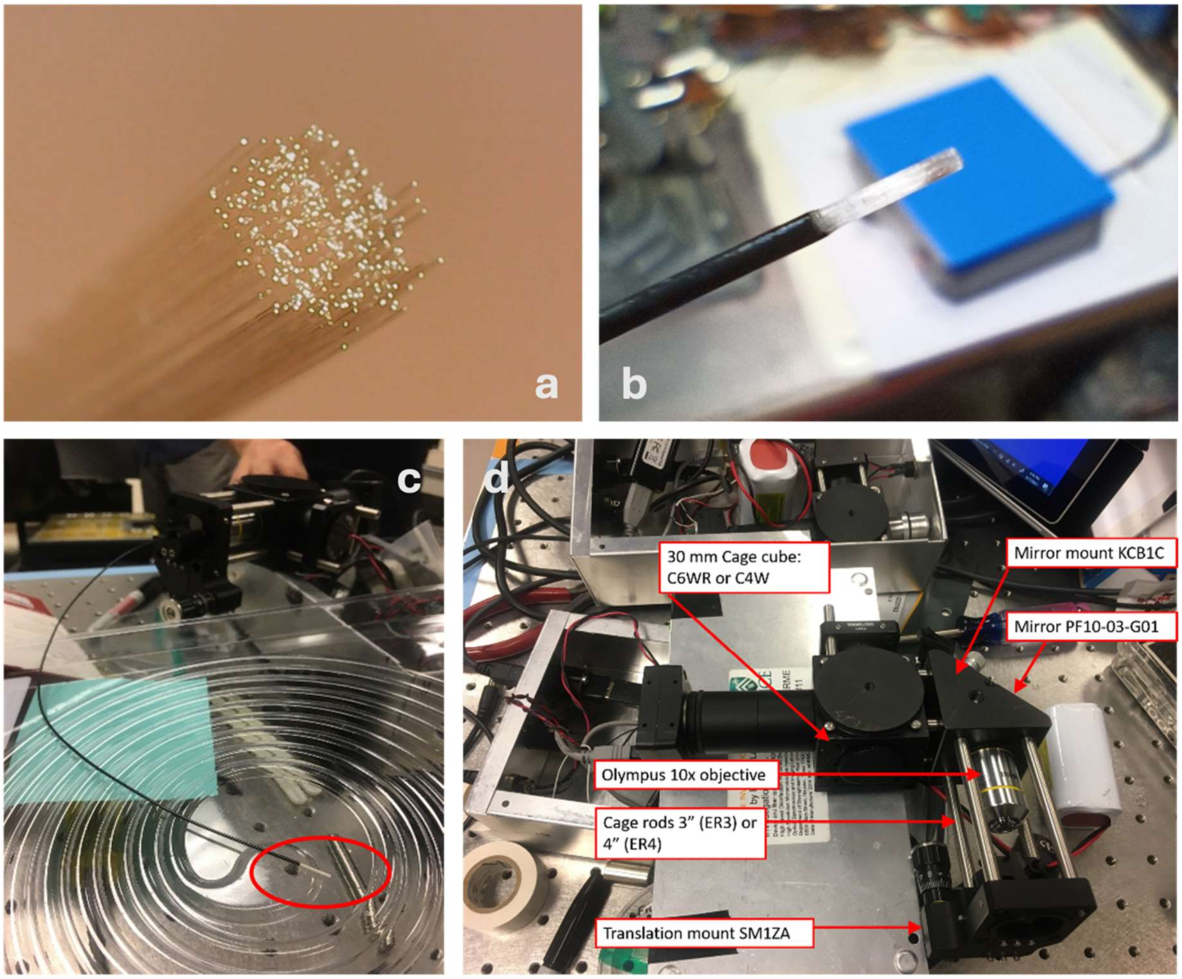

Figure S2. (a) An open-end of an optical fiber brush cut off before polishing. (b) Temporary compressed and polished end of the unfused optical fiber bundle. (c) The polished open-end of the optical fiber brush prepared for image data acquisition and connected to the HRME device. (d) The image acquisition add-on to the HRME device is schematically labeled and presented in the picture.

After cutting off the fiber bundle, the open-ends were custom-made polished, and added as an image acquisition add-on (Figure S2) to the HRME device described in detail previously [18,19].

The overall schematic of the imaging system is shown in Figure S3. One can see that the unfused-end of the optical fiber brush collected data from a potential sample and the fused-end of an optical fiber bundle is projecting data to the camera. Image data received from two types of optical fibers are shown in Figure S3 b and c.

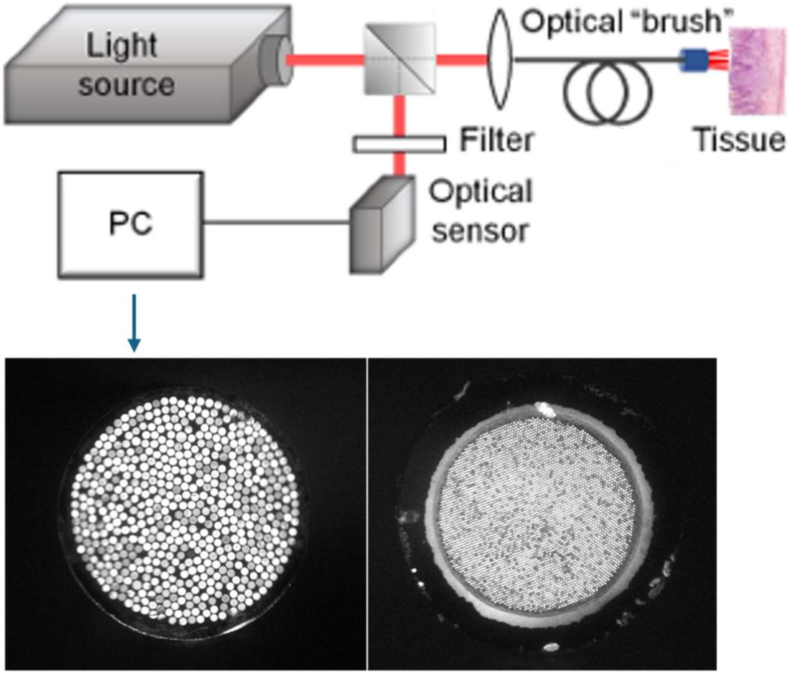

Figure S3. Schematic of the HRME imaging system and an endoscopic expandable optical fiber brush to collect image data. The data received in the camera are demonstrated for two types of fiber bundles (left: single fiber ∅ 29 µm, outer fiber bundle ∅ 600 µm; right: single fiber ∅ 6 µm, outer fiber bundle ∅ 500 µm).

The next step of the prototyping is the development of a miniature mechanism[9] that could expand the optical fiber bundle brush at the distal end of an endoscope in a controlled manner. Such a mechanism will allow close to equal distribution of single fibers across sections of FOV in the examined area.

The optical fiber brush prototyping is currently limited by the availability of commercial non-fused optical fiber bundles with required diameters of single fibers and outer diameters of fiber bundles. Having more optical fiber brush prototypes will enable more accurate safety testing for its clinic application. The development of a radial model for deep-learning super-resolution sparse image reconstruction will be the further improvement of the proposed concept.
